# A Cross-Sectional Study on Prevalence and Predictors of Burnout among a Sample of Pharmacists Employed in Pharmacies in Central Italy

**DOI:** 10.1155/2019/8590430

**Published:** 2019-12-24

**Authors:** Carmela Protano, Simone De Sio, Vittoria Cammalleri, Roberta Noemi Pocino, Stefano Murano, Roberto Perri, Giuseppe Buomprisco, Maria De Giusti, Matteo Vitali

**Affiliations:** ^1^Department of Public Health and Infectious Diseases, University of Rome “La Sapienza”, P.le Aldo Moro 5, 00185 Rome, Italy; ^2^Specialty School of Occupational Medicine—Research Unit of Occupational Medicine, University of Rome “La Sapienza”, P.le Aldo Moro 5, 00185 Rome, Italy

## Abstract

Burnout is defined as an occupational phenomenon linked to chronic workplace stress that has not been successfully managed and included among the factors influencing health status or contact with health services. Although several studies were performed for assessing this phenomenon, there is a lack of data on the prevalence of burnout and associated predictors, due to different definitions of the syndrome and heterogeneity of assessment methods. One of the well-known evidences on burnout is related to the highest risk professions, which include policemen, firemen, teachers, psychologists, medical students, nurses, physicians, and other health professionals, such as pharmacists. *Objective*. The aims of the present study were to (1) assess the occurrence of burnout syndrome among a sample of pharmacists employed in public and private pharmacies located in Rome province (Latium Region; central Italy); (2) evaluate the role of some potential predictors for the development of the syndrome. *Materials and Methods*. A questionnaire elaborated ad hoc was administered online to 2,000 members of the Association of Professional Pharmacists of Rome and its province and employed in public or private pharmacies. The questionnaire included the 14-item Shirom–Melamed Burnout Measure (SMBM) tool and questions on demographic characteristics and working conditions. *Results*. Physical exhaustion was the burnout dimension with the highest score; besides, approximately 11% of the studied pharmacists were categorized as having clinically relevant burnout levels (≥4.40). Several of the investigated variables significantly influenced the single burnout dimensions at the univariate analyses; multivariate analyses demonstrated that alcohol consumption and workplace location have a significant independent role on the overall SMBM index, while working time significantly influences clinically relevant burnout level. *Conclusions*. The results revealed that pharmacists are at risk of burnout, and thus, it is necessary to perform specific preventive intervention for managing this occupational threat.

## 1. Introduction

According to Maslach's definition, Burnout Syndrome is characterized by emotional exhaustion, depersonalization, and reduced professional effectiveness which can occur in subjects who, in their professional life, work in contact with people [1]. This syndrome is characterized by three dimensions: fatigue, cynical attitude, and inefficacy both related to one's job [2].

Recently, the World Health Organization updated its definition of burnout that will go into effect in 2022, classifying it as an “occupational phenomenon” linked to “chronic workplace stress that has not been successfully managed” and including it among the “factors influencing health status or contact with health services” [3].

Although several epidemiological studies were performed in order to evaluate this phenomenon, there is a lack of data on the prevalence of burnout, due to different definitions of the syndrome and heterogeneity of assessment methods. This lack of evidence was also pointed out by a recent review, demonstrating a remarkable variability in published prevalence of burnout (ranging from 0% to 80.5%) and highlighting the necessity to develop a common definition for burnout and standardized measurement tools [4].

The most exposed professions to burnout are policemen, firemen, teachers, psychologists, medical students, nurses, physicians, and other health-care workers [4–13]. A recent study, carried out on a sample of pediatricians, showed a high degree of exhaustion in at least one of the three burnout dimensions for 49% of the enrolled physicians; besides, nearly 90% of them had thought of leaving their profession because of their level of work stress [6]. Another observational study on Italian hospital nurses demonstrated high levels of emotional exhaustion (55%) and depersonalization (42%). The three burnout dimensions and the three work engagement dimensions showed a close and meaningful correlation (*p* ≤ 0.01) [14]. Also health professions students are at high risk of burnout. Indeed, a study conducted on a large sample of American medical students showed an increase of distress from 2% to 12% and burnout from 17% to 38% from matriculation through after the residency match, demonstrating the high risk of developing burnout during medical school [15].

Over time, several studies reported some significant predictors of psychosocial risk such as age, gender, work-related factors, etc [16, 17].

Among helping professions, pharmacists are a category of workers undervalued with respect to the risk of developing burnout. In particular, those employed in pharmacies have a continuous contact with ill or suffering people and may play a key role in the early assessment of medical errors in drug prescription as well as in supporting the choice and correct use of drugs and medications [18]. A research carried out in 2016 in the United States, assessing the job satisfaction degree in a large sample of pharmacists, showed that 72.5% of studied workers were satisfied with their work, but 63.4% noticed an increased work stress during the course of the years [19]. Another survey, performed by Durham et al. in the same year and studied area on a sample of health-system pharmacists, showed that 53.2% of participants presented a high degree of burnout on the emotional fatigue scale. Furthermore, 8.5% of the sample had a positive burnout in emotional fatigue, personal fulfilment, and depersonalization scales [20]. In addition, also pharmacy students are at risk of burnout; in this context, a very recent study reported that a large percentage of pharmacy students declared that they had a significant burnout level [21]. Thus, pharmacists can be considered at high risk of burnout and need further research for identifying the prevalence of the phenomenon and its predictors, in order to define appropriate preventive strategies [22].

The aims of the present study were to (1) assess the occurrence of burnout syndrome among pharmacists employed in public and private pharmacies located in the metropolitan area of Rome and (2) evaluate the role of some potential predictors for the development of the syndrome.

## 2. Materials and Methods

### 2.1. Study Design and Population

The present survey, consisting of a cross-sectional study, was performed online among all the members of the Association of Professional Pharmacists of metropolitan area of Rome, employed in public or private pharmacies. The study protocol was approved by the Board of the Association.

The questionnaire included the 14-item Shirom—Melamed Burnout Measure (SMBM) tool [23], several questions on demographic characteristics of the participant, and some about working conditions. In particular, demographic data referred to gender, weight, and height for calculating Body Mass Index (BMI), nationality, smoking, and drinking habits, with details about contract typology (permanent or temporary contracts), job seniority, night shifts, workplace location, night shifts, and workplace location. The validity and reliability of the 14-item SMBM tool in adult samples were recently examined by Schilling et al. [24]; they demonstrated that it is a valid and reliable tool to assess burnout in both male and female adults and in different professional populations. In order to assess the reliability of the questionnaire among pharmacists, before starting the present study, the questionnaire was preliminarily administered to 30 pharmacists; the collected data were used to calculate Cronbach's *α* for estimating the reliability of test items. Cronbach's *α* resulted >0.80 for all of the investigated domains.

### 2.2. Covariates

All the responses were coded and entered in a database created “ad hoc.”

The participants were asked to respond to the 14-item SMBM according to the 7‐point scale (never or almost never, very infrequently, quite infrequently, sometimes, quite frequently, very frequently, and always or almost always). Total points were recorded and the scores for each domain (physical exhaustion, emotional exhaustion, and cognitive weariness) were calculated. The corresponding values for each domain were added together and then divided by 3 to obtain the overall SMBM Index. Participants were considered as having clinically relevant burnout level when the overall SMBM Index resulted ≥4.40 [25]. Thus, we recoded the variable as “clinically relevant burnout level”: No when the overall SMBM Index was <4.40 and Yes when the overall SMBM Index was ≥4.40.

### 2.3. Statistical Elaboration

Statistical analysis was performed using IBM SPSS Statistics 25 software (IBM Corp., Armonk, NY, USA). Data obtained from the survey were entered into a database elaborated. Continuous variables were reported as arithmetic mean (AM) ± Standard Deviation (SD), whereas categorical variables were reported as absolute and relative frequencies.

Relationships between each of the three dimensions of burnout or overall SMBM index and demographic characteristics, smoking and alcohol consumption habits, and the investigated working conditions were assessed by the use of Mann–Whitney tests. The association between the scores of the four variables (the single three dimensions and the overall index) and age or BMI was evaluated by the use of Spearman's correlation coefficients. *p* value ≤0.05 was considered statistically significant for both statistical tests. Then, we focused the attention on the variable “clinically relevant burnout level” (considering the score 4.40 as the cut-off) and evaluated its association with the demographic characteristics, smoking and alcohol consumption habits, and the investigated working conditions by the use of chi square test.

Finally, multivariate analyses were performed in order to evaluate the independent role of the variables that resulted influencing significantly the likelihood of having burnout syndrome. For this purpose, we used both the overall SMBM index and the clinically relevant burnout level as the dependent variable. In particular, first we performed a multivariate linear regression analysis to assess the independent contribution of some variables on the overall SMBM index. This analysis was carried out using a significance level of 0.05 for entry and 0.10 for removal from the model. The significance level for the analysis was *p* ≤ 0.05 (two-tailed). The “goodness of fit” of the model was assessed using *R*^2^ statistics. Then, we run a multivariate logistic regression analysis to assess the independent role of some variables on the clinically relevant burnout level. For this purpose, we used a backward elimination procedure and *p* ≤ 0.05 (two tailed) was used as the threshold for removing a variable from the model.

## 3. Results

Table 1 shows the relevant characteristics of the studied population.

In total, 469 pharmacists out of about 2000 members of the Association of Professional Pharmacist of metropolitan area of Rome and employed in a public or private pharmacy took part in the present survey (24% response rate). Regarding the characteristics of the study sample, approximately two-thirds of participants were females; the age ranged from 25 to 68 years, and most of participants were Italian (98.5%). Consequently, we did not consider this variable in the statistical elaboration. With respect to lifestyles, more than 80% of the pharmacists did not smoke, but more than half participants admitted consuming alcohol. With regard to the work characteristics, most of the participants had a permanent job and were full-time employees and not performing night shifts. Just over half of the participants had been employed for more than 10 years and almost half of them had >5 hours of overtime/month. In addition, workplace was located within Rome municipality for about 75% of the cases. With respect to the three dimensions of burnout, the highest score was found for physical exhaustion (AM = 4.18); this result is probably related to pharmacist's sales activity, that is very tiring in many cases, standing up for most of the working hours. Besides, approximately 11% of the studied pharmacists can be categorized as having clinically relevant burnout levels (≥4.40).

Table 2 reports the association between the three dimensions of burnout or the levels of overall SMBM index and gender or some lifestyle habits (smoking habits and alcohol consumption).

As shown in Table 2, alcohol consumption habit analysis demonstrated that there is a significant higher risk of emotional exhaustion for those who consume alcohol (AM ± SD = 2.00 ± 2.00) with respect to those had not this habit (AM ± SD = 1.66 ± 1.66), statically significant (*p*=0.004). Likewise, subjects consuming alcohol present a significantly higher overall SMBM index with respect to the others (*p*=0.027).

Table 3 shows the association between some working characteristics (workplace location, working position, and type of working contract) and the three dimensions of burnout or overall index.

Based on data reported in Table 3, working outside Rome municipality significantly increases the scores of physical exhaustion and overall SMBM index. Besides, being employed in a pharmacy significantly increases the risk of physical exhaustion with respect to the other roles (*p* value = 0.006).

Table 4 reports the association between working characteristics in terms of time (job seniority, part time or full time, night shifts, and average overtime) and the three dimensions of burnout or overall index.

As reported in Table 4, job seniority significantly influences the score of burnout dimensions; indeed, pharmacists who have worked for more than 10 years have a higher level of cognitive weariness (*p*=0.017) and emotional exhaustion (*p*=0.012) with respect to the others.

Table 5 shows Spearman's rank correlation coefficients found between age or BMI and the three investigated dimensions of burnout (physical exhaustion or cognitive weariness or emotional exhaustion, respectively) or overall SMBM index.

Age and cognitive weariness present a significant positive correlation (*p* value = 0.0028).

Table 6 shows the univariate analyses on the association between the variable “clinically relevant burnout level” and the demographic characteristics, smoking, and alcohol consumption habits and the investigated working conditions.

According to data reported in Table 6, average overtime and working time are significant predictor of clinically relevant burnout level.

Tables 7 and 8 report the results of multivariate analyses performed to evaluate the independent role of the variables that resulted significantly influencing overall SMBM index (linear regression analysis, Table 7) and “clinically relevant burnout level” (logistic regression analysis, Table 8).

The results of linear regression analysis confirm totally the results of univariate analyses, evidencing that both alcohol consumption and workplace location have a significant independent role on the overall SMBM index. In particular, alcohol consumption and work at Rome municipality increase the risk of burnout syndrome for pharmacists. In contrast, logistic regression analysis confirmed just partially the results of univariate analyses. Indeed, the only variable that resulted influencing the “clinically relevant burnout level” was the working time.

The selected final multivariate models explained a small percentage of their variance; explanation to this result can be related to other individual determinants of the risk of burnout determining the variability but not considering in this study.

## 4. Discussion

The present study was performed in order to investigate the frequency of burnout and its predictors among pharmacists employed in public and private pharmacies. The first relevant finding was associated to the role of gender on the development of burnout. Our analysis demonstrated no significant differences in burnout levels (both for the three specific dimensions and the overall index) of the investigated men and women, according to previous research performed among a sample of community pharmacists in Ankara [26]. In contrast, other studies demonstrated that men suffer significantly more of burnout syndrome than women and that women are at higher risk of mental disorders, depression, anxiety, and psychosomatic illnesses and men seem to suffer more from higher levels of stress [27, 28]. In addition, previous researches evidenced that female physicians present a lower risk of depersonalization and a higher risk of emotional exhaustion with respect to males [29, 30]. Also, a nationwide mail survey performed among a sample of the American Pharmaceutical Association membership demonstrated higher level of burnout in women with respect to men pharmacists [31]. Smoking habit analysis revealed no significant differences in burnout levels between smokers and no smokers, according to a study among French pharmacists [27]. In contrast, alcohol consumption significantly increases the scores of emotional exhaustion and of overall SMBM index. This phenomenon could be related to the well-known emotion-altering effect of alcohol. Accordingly, Balayssac et al. [27] found that, even if alcohol consumption in general was not associated with burnout, drinking amounts of alcohol above the World Health Organization (WHO) limits was related to the severity of burnout for males.

Besides, we found that workplace location significantly influenced the risk of burnout: working in Rome (an urban city with a high urbanization degree) significantly increases the levels of both physical exhaustion and overall SMBM index, according to a study that confirmed the presence of a higher burnout severity among a sample of pharmacists working in a large urban area [27]. This association was proved also for other healthcare professions [30] and for school teachers [32]. In particular, Saijo et al. [30] demonstrated greater job demands, less job control, and greater exhaustion among urban hospital physicians despite the rural hospital ones. Likewise, Dombrovskis et al. [32] evidenced higher levels of professional burnout scales (emotional exhaustion, depersonalization, and personal accomplishment) among urban school teachers with respect to rural ones.

In addition, in our study population, the pharmacists most exposed to physical exhaustion were those who played the role of employee compared to those who held the role of holder, director or other management roles. This result is not surprising, as the employees in a pharmacy experience a relevant physical load of work, especially in larger pharmacies [33].

With regard to the type of working contract, scientific literature addressed that psychosocial risks, including burnout, can be influenced by job insecurity and fixed-term contracts [34, 35]. In contrast, we did not find any difference between pharmacists with temporary job respect to those with permanent job. Indeed, in Italy, fixed-term contracts of individuals employed in public or private pharmacies are very often converted into permanent contracts. Likewise, the category of Italian pharmacists does not perceive job insecurity.

Significant differences were found between job seniority and cognitive weariness or emotional exhaustion. Probably, job seniority is directly proportional to the increase of the risk to develop burnout. These results are in contrast with recent studies; Calgan et al. [26] reported that pharmacists who worked for less than 10 years have a higher level of emotional exhaustion and depersonalization than those working for more than 10 years; Durham et al. [20] reported that pharmacists working for less time were more exposed to burnout. In these studies, experience seems to be a protective factor for burnout risk.

A significant positive correlation was found between cognitive weariness and age. This result should explain the relationship we found between job seniority and cognitive weariness. In practice, older pharmacists, who worked for more years, are at higher risk of burnout. In contrast with our findings, Calgan et al. [26] found that younger pharmacists are at higher risk of emotional exhaustion and depersonalization with respect to older ones. Similarly, Jones et al. [2] evaluated factors associated with burnout among a sample of hospital clinical pharmacy practitioners in United States and demonstrated that age was shown to be protective against burnout. These results can be explained considering that younger pharmacists have less work experience, and thus, they presumably present a greater emotional load during working activities compared to older ones. Indeed, older pharmacists have spent several/many years of their life in contact with patients, and probably, they have “accumulated” greater fatigue and responsibilities. Besides, cognitive weariness is also certainly influenced by the physiological cognitive impairment that occurs over the years. In addition to these considerations, the results of another study on Canadian workers demonstrated the complexity of the association between gender, age, and the risk of burnout syndrome; indeed, the cited study evidenced that age was associated with emotional exhaustion and total burnout through a nonlinear relationship, while the same variable was associated to cynicism and reduced professional efficacy through a linear relationship. In addition, the risk of burnout was inversely related to age in the group of men, while it resulted “bimodal” in the group of women [36].

The present study had some limitations. First, this is a cross-sectional study and, consequently, we cannot make general considerations on causal relationships or temporal order; however, the investigated predictors such as alcohol consumption or workplace locations were stable indicators of lifestyle habits or working conditions during the study period. Also, the participation rate was not so high. The high percentage of nonresponse can be attributed both to the scarce attitude of pharmacists to check emails, especially older pharmacists, and to the underestimation of burnout syndrome. Besides, the great variability of demographic characteristics, lifestyles, and working conditions of the study sample allows to consider it as representative of the pharmacists employed in the private and public pharmacies of Rome and province.

## 5. Conclusions

Our results demonstrate that several of the investigated variables significantly influence the single burnout dimensions at the univariate analyses. In addition, multivariate analyses demonstrated that alcohol consumption and workplace location have a significant independent role on the overall SMBM index, while working time significantly influences the “clinically relevant burnout level”. However, the results of multivariate models evidenced that other individual confounding/interfering factors of the risk of burnout in pharmacists should be investigated. Finally, our findings revealed that pharmacists are at risk of burnout, and thus, it is necessary to perform specific preventive intervention for managing this occupational risk.

## Figures and Tables

**Table 1:** Characteristics of the studied population.

		*n* (%) or AM ± SD
Gender *n* (%)	M	121 (25.8)
	F	348 (74.2)

Nationality *n* (%)	Italian	463 (98.5)
	Not Italian	6 (1.5)

Smoking habit *n* (%)	No	384 (81.7)
	Yes	85 (18.3)

Alcohol consumption *n* (%)	No	184 (39.2)
	Yes	285 (60.8)

Type of working contract *n* (%)	Temporary job	73 (15.5)
	Permanent job	396 (84.5)

Working position *n* (%)	Employee	362 (77.2)
	Other	107 (22.8)

Night shifts *n* (%)	No	439 (93.4)
	Yes	30 (6.6)

Workplace location *n* (%)	Rome municipality	352 (74.9)
	Outside Rome municipality	117 (25.1)

Job seniority *n* (%)	<10 years	206 (43.8)
	≥10 years	263 (56.2)

Average overtime *n* (%)	0–5	244 (51.9)
	>5	225 (48.1)

Working time *n* (%)	Part time	149 (31.7)
	Full time	320 (68.3)

Age (AM ± SD)		(42.6 ± 10.8)

Body mass index (BMI) (AM ± SD)		(22.6 ± 4.2)

Phisycal exhaustion		4.18 ± 1.50

Cognitive weariness		2.63 ± 1.26

Emotional exhaustion		2.27 ± 1.29

Overall SMBM index		3.03 ± 1.10

Clinically relevant burnout level *n* (%)	No	420 (89.5)
	Yes	49 (10.5)

**Table 2:** Association between scores (0–7 points scale) for the three dimensions of burnout or the levels of overall SMBM index and gender or lifestyle habits (smoking habits and alcohol consumption).

	Gender		*p* value^a^	Smoking habits		*p* value^a^	Alcohol consumption		*p* value^a^
	Male	Female		No	Yes		No	Yes	
Physical exhaustion	4.00	4.16	NS	4.08	4.16	NS	4.00	4.16	NS
Cognitive weariness	2.60	2.4	NS	2.40	2.40	NS	2.40	2.60	NS
Emotional exhaustion	2.00	2.00	NS	2.00	2.00	NS	1.66	2.00	0.004
Overall SMBM index	3.02	2.89	NS	2.91	2.91	NS	2.78	3.00	0.027

^a^ *p* values were assessed by the use of Mann–Whitney tests. NS = not significant.

**Table 3:** Association between scores (0–7 points scale) for the three dimensions of burnout or the levels of overall SMBM index and some working characteristics.

	Workplace location		*p* value^a^	Working position		*p* value^a^	Type of working contract		*p* value^a^
	Rome municipality	Outside Rome municipality		Employee	Other		Temporary job	Permanent job	
Physical exhaustion	4.16	3.83	0.003	4.16	3.83	0.006	4.00	4.16	NS

Cognitive weariness	2.60	2.40	NS	2.50	2.4	NS	2.60	2.40	NS

Emotional exhaustion	2.00	1.66	NS	2.00	1.6	NS	2.00	2.00	NS

Overall SMBM index	2.97	2.67	0.027	2.95	2.71	NS	2.91	2.91	NS

^a^ *p* values were assessed by the use of Mann–Whitney tests. NS = not significant.

**Table 4:** Association between scores (0–7 points scale) for the three dimensions of burnout or the levels of overall SMBM index and some working characteristics in terms of time.

	Job seniority		*p* value^a^	Night shifts		*p* value^a^	Average overtime		*p* value^a^	Working time		*p* value^a^
	≤10 years	>10 years		No	Yes		0–5 hours	>5 hours		Part time	Full time	
Physical exhaustion	4.00	4.16	NS	4.16	4.08	NS	4.08	4.16	NS	4.00	4.16	NS
Cognitive weariness	2.40	2.60	0.017	2.40	2.80	NS	2.60	2.40	NS	2.40	2.60	NS
Emotional exhaustion	1.66	2.00	0.012	2.00	2.00	NS	2.00	2.00	NS	2.00	2.00	NS
Overall SMBM index	2.83	3.03	NS	2.9	3.02	NS	2.92	2.86	NS	2.84	2.96	NS

^a^ *p* values were assessed by the use of Mann–Whitney tests. NS = not significant.

**Table 5:** Spearman's rank correlation coefficients between age or BMI and physical exhaustion or cognitive weariness or emotional exhaustion, or overall SMBM index.

	Age		BMI	
	Correlation coefficient	*p* value	Correlation coefficient	*p* value
Physical exhaustion	−0.018	0.692	0.010	0.846
Cognitive weariness	0.102	**0.028**	0.066	0.190
Emotional exhaustion	0.086	0.062	0.083	0.097
Overall SMBM index	0.057	0.215	0.070	0.164

**Table 6:** Association between “clinically relevant burnout level” and demographic characteristics, smoking and alcohol consumption habits, and working conditions.

		Clinically relevant burnout level		
		No *n* (%)	Yes *n* (%)	*p* value
Gender	M	104 (86.0)	17 (14.0)	0.150
	F	313 (89.9)	35 (10.1)	

Smoking habit	No	338 (88.0)	46 (12.0)	0.130
	Yes	79 (92.9)	6 (7.1)	

Alcohol consumption	No	165 (89.7)	19 (10.3)	0.396
	Yes	252 (88.4)	33 (11.6)	

Type of working contract	Temporary job	67 (91.8)	6 (8.2)	0.266
	Permanent job	350 (88.4)	46 (11.6)	

Working position	Employee	318 (87.8)	44 (12.2)	0.117
	Other	99 (92.5)	8 (7.5)	

Night shift	No	390 (88.8)	49 (11.2)	0.569
	Yes	27 (90.0)	3 (10.0)	

Workplace location	Rome municipality	312 (88.6)	40 (11.4)	0.445
	Outside Rome municipality	105 (89.7)	12 (10.3)	

Job seniority	<10 years	189 (91.7)	17 (8.3)	0.056
	≥10 years	228 (86.7)	35 (13.3)	

Average overtime	0–5	224 (91.8)	20 (8.2)	**0.027**
	>5	193 (85.8)	32 (14.2)	

Working time	Part time	140 (94.0)	9 (6.0)	**0.011**
	Full time	277 (86.6)	43 (13.4)	

**Table 7:** Significant predictors of overall SMBM index in forward multiple linear regression models.

Independent variable	B (regression coefficient)	Standard error	*β* (regression standardized coefficient)	*p* value	Adjusted *R*^2^
Constant	2.945	0.085		<0.001	
Alcohol consumption	0.230	0.103	0.103	0.026	0.015
Workplace location	−0.232	0.117	−0.091	0.047	

Linear regression final model (forward method); variables included in the model: gender (female vs. male), age (as continuous variable), alcohol consumption (No vs. Yes), and workplace location (Rome municipality vs. outside Rome municipality).

**Table 8:** Logistic regression analyses of “clinically relevant burnout level” among studied pharmacists.

	Adjusted^a^ OR (95% CI)	*p*
Average overtime		
0–5	Reference	0.092
>5	1.67 (0.91, 3.03)	

Working time		
Part time	Reference	0.030
Full time	2.20 (1.03, 4.68)	

OR: odds ratio; 95% CI: 95% confidence interval. ^a^Adjusted for all the four variables reported in the table as well as for gender and age.

## Data Availability

The dataset used to support the findings of this study is restricted in order to protect the privacy of the studied individuals. Data are available from Matteo Vitali (matteo.vitali@uniroma1.it) for researchers who meet the criteria for access to confidential data.
